# The variable, task-dependent effects of sleep deprivation across working life still need our attention: in the laboratory and beyond

**DOI:** 10.1093/sleep/zsaf161

**Published:** 2025-06-09

**Authors:** Amy C Reynolds, Kelly Sansom, Andrew Vakulin, Robert J Adams

**Affiliations:** Flinders Health and Medical Research Institute (Sleep Health), College of Medicine and Public Health, Flinders University, Bedford Park, South Australia, Australia; Flinders Health and Medical Research Institute (Sleep Health), College of Medicine and Public Health, Flinders University, Bedford Park, South Australia, Australia; Flinders Health and Medical Research Institute (Sleep Health), College of Medicine and Public Health, Flinders University, Bedford Park, South Australia, Australia; Flinders Health and Medical Research Institute (Sleep Health), College of Medicine and Public Health, Flinders University, Bedford Park, South Australia, Australia

As the global population of older adults grows, many are remaining in the workforce longer [1, 2]. Understanding how sleep deprivation affects this population is essential, particularly in high-risk industries or in occupations that may impact sleep timing and opportunity. While more than 20 years of evidence now shows us that healthy young adults exhibit substantial inter-individual and task dependent variability in their functional vulnerability to sleep loss [3-5], less is known about these patterns in older adults. Early evidence suggested that older individuals may be more tolerant of sleep loss [6] relative to the vulnerability observed in younger adults [6, 7], but whether this perceived resilience holds across different tasks remained unclear.

In this issue of SLEEPJ, Francis-Pester et al. [8] expand our understanding of inter-individual differences in response to sleep deprivation, with a focus on age-related differences within younger and older working-age adults. They report nuanced differences in functional impairment (wake electroencephalography (EEG), ocular, attention, and vigilant attention) for some younger adults, relative to their older counterparts. Recognizing that the sample size is modest, the authors highlight that inter-individual differences in response to sleep deprivation between young adults were more variable than responses between older adults. This was particularly apparent in younger female participants and was evidenced by greater sleep initiation events, characterized by slow eye movements, microsleeps, and higher EEG delta power. This builds on previous findings that younger adults were more impaired than older adults on a closed-loop driving track after total sleep deprivation [9], to better understand the underlying drivers of impairment in younger versus older adults.

The framing of sleep-initiation versus non-sleep-initiation events offers a valuable lens for future studies to explore where and how age-related vulnerabilities may manifest in real-world settings. Understanding how these patterns translate to performance in specific job roles that rely on maintenance of vigilant attention (e.g. commercial drivers) versus more complex executive function where deficits were observed in older adults is an important next step.

While inter-individual variability is both stable and replicable during sleep deprivation [5], we still have a limited picture of what constitutes a “vulnerable phenotype” for sleep deprivation, particularly in ecologically valid contexts and in populations with preexisting suboptimal sleep or sleep disorders. Notably, even in the current study with a highly screened “best-case” sleep group, some individuals, especially younger adults, exhibited marked vulnerability to sleep deprivation. This raises an important question: what happens to performance under sleep deprivation conditions when habitual sleep is already compromised?

From the limited available laboratory evidence, we know that middle-aged people with obstructive sleep apnea (OSA) are significantly more vulnerable to the effects of sleep restriction on driving performance compared to age-matched healthy sleepers [10]. Acute sleep deprivation has also been shown to result in greater impairments in vigilant attention for snorers versus non-snorers [11]. The inter-individual variability in functional response (driving performance and vigilant attention) to sleep restriction in people with OSA is greater relative to healthy sleepers and is driven predominantly by a minority of vulnerable individuals at high risk [12]. The impact of acute total or partial sleep deprivation in the laboratory on young and older adults with other sleep disorders (e.g. insomnia and circadian rhythm sleep–wake disorders) remains a significant gap in our knowledge.

This is problematic, as sleep disorders are prevalent across working-aged adults; 20 per cent of 22-year-olds report clinically significant sleep concerns (insomnia being the highest prevalence) [13], yet fewer than one in five are diagnosed [14]. By middle age, this increases to 43 per cent (with OSA more prevalent by this point) [15], and seems to rise modestly again into older adulthood—especially for obstructive sleep apnea and insomnia [16]. Disorder prevalence in both younger and older adults highlights the need to better understand how sleep loss manifests and impacts functioning across different life stages, particularly when sleep is impacted by comorbid health and other lifestyle factors.

Much of what we know about sleep deprivation comes from laboratory studies involving healthy sleepers, which may not reflect the full spectrum of real-world sleep challenges, including sleep disorders. Consequently, while laboratory studies of sleep deprivation in healthy sleepers have been instrumental in identifying age-related and individual differences in vulnerability to sleep loss, they likely underestimate the real-world impacts and complexity of sleep disruption, sleep loss, and aging. This is particularly relevant given the functional consequences observed in both age groups: in young adults, sleep loss is linked to academic underperformance [17], mood [18], and risk-taking [19]; in older adults, it contributes to cognitive decline [20] and falls [21].

As we consider the translational implications of highly variable sleep-initiation events during sleep deprivation in young adults, we are reminded that while task-dependent impairment is likely nuanced according to age, there remains more to learn about impairment with acute sleep deprivation beyond our “healthy” sleepers. Casting a wider net to understand the consequences for “non-healthy” sleepers in laboratory and controlled field studies of sleep deprivation and their impacts on the observed variability and vulnerability are essential. Through this, we can begin to explore more tailored approaches to managing sleep loss, with a view to more targeted advice about identifying, preparing for, and responding to acute periods of total or partial sleep deprivation.
